# 201. Frequency of *Pseudomonas aeruginosa* (PA) Discrete Inner Colonies and Comparison of Minimal Inhibitory Concentration (MIC) Values between Parent and Inner Colony Isolates Following Fosfomycin Disk Diffusion (DD) Testing

**DOI:** 10.1093/ofid/ofab466.201

**Published:** 2021-12-04

**Authors:** Dina Zheng, Morgan L Bixby, Elizabeth C Smith, Jadyn C Anderson, Phillip J Bergen, Cornelia B Landersdorfer, Elizabeth B Hirsch

**Affiliations:** 1 University of Minnesota College of Pharmacy, Minneapolis, MN; 2 Monash University Centre for Medicine Use and Safety, Melbourne, Victoria, Australia

## Abstract

**Background:**

Fosfomycin combination therapy is a potential approach for treatment of multidrug-resistant (MDR) PA infections despite a lack of approved susceptibility breakpoints for this organism. While DD testing is commonly used for fosfomycin, growth of discrete inner colonies (IC) within the zone of inhibition has been observed for multiple organisms following DD. Criteria recommended by CLSI and EUCAST are contradictory for interpreting these inner colonies. We therefore sought to determine the frequency of inner colonies and MIC differences between PA parent-inner colony pairs from an international isolate collection.

**Methods:**

A convenience collection of 198 clinical PA isolates from three U.S institutions (n = 82), two Australian institutions (n = 72), and the CDC & FDA Antibiotic Resistance Isolate Bank (n = 44) were included. Fosfomycin MIC values were determined in duplicate on separate days by DD and broth microdilution (BMD) testing. For parent isolates with discrete IC observed during DD, IC isolates were subcultured and MIC values were determined and then compared to their corresponding parent isolates. MIC values were interpreted using CLSI *Escherichia coli* (EC) breakpoints (susceptible: MIC ≤ 64 μg/mL, intermediate: MIC = 128, resistant: MIC ≥ 256 μg/mL).

**Results:**

Parent isolate BMD MIC values ranged from < 4 to > 256 μg/mL while IC isolate BMD MIC values ranged from 128 to > 1024 μg/mL. MIC_50/90_ values were 128/256 μg/mL and > 1024/ > 1024 μg/mL for the parent and IC isolates, respectively. A high frequency of 45% (89/198) of parent isolates displayed discrete IC which also demonstrated a higher frequency of resistance (97.8%) compared to the parent isolates (23.7%).

**Conclusion:**

IC MIC values were higher overall compared to parent MIC values, with an average fold difference of ~18 between the parent-inner colony pairs. The frequency of IC found in this study (45%) is considerably higher than previously observed in EC clinical isolates. These data highlight the need to further investigate the importance of these IC and warrant caution for extrapolation of EC breakpoints for fosfomycin susceptibility testing against PA.

**Disclosures:**

**All Authors**: No reported disclosures

